# Promoting the Well-Being of Older People in Ethiopia: Lost Opportunities Due to the Poverty of Policy

**DOI:** 10.1093/geroni/igad120

**Published:** 2023-10-19

**Authors:** Margaret E Adamek, Messay Gebremariam Kotecho, Abraham Zelalem Teshome

**Affiliations:** School of Social Work, Indiana University, Indianapolis, Indiana, USA; School of Social Work, Addis Ababa University, Addis Ababa, Ethiopia; School of Social Work, Addis Ababa University, Addis Ababa, Ethiopia; Department of Social Work and Community Development, University of Johannesburg, Johannesburg, South Africa; School of Social Work, Indiana University, Indianapolis, Indiana, USA

**Keywords:** African older adults, Ageism, Aging policy, Ethiopia

## Abstract

The world’s population is aging with the fastest growth in the older population projected to take place in Africa. In this article, we present the *challenges* of the growing older population in Ethiopia, outline some key *changes* that are needed to address those challenges, and consider the *opportunities* that can come about when older adults’ basic needs are met and they are supported in contributing to their communities. Older adults in Ethiopia are faced with multidimensional challenges that call for collaborative efforts from different stakeholders at local, regional, and national levels. However, some measures should be given the utmost priority: combating negative attitudes toward older people, strengthening geriatric/gerontology and social work education and research, and developing aging-specific policies and services. Although the challenges faced by older adults in Ethiopia seem like a problem of aging compounded by poverty, the root source of the problem is a *poverty of policy* fueled by ageism. If income support and appropriate health care was provided to older adults throughout Ethiopia, health and well-being in late life would improve, food and housing insecurity among older adults would lessen, and all Ethiopians could anticipate a dignified late life.

**Translational Significance:** In Ethiopia, as in many sub-Saharan nations, the life circumstances of most older adults are deteriorating. Drawing from recent literature and authors’ own observations, we outline the challenges faced by Ethiopians as they age, the changes that are needed to address the challenges, and opportunities that can come about if older adults are provided with basic support such as social protection and geriatric health care. The government of Ethiopia must take meaningful action in honoring older adults by welcoming their contributions and providing social protection to uphold their human rights.

“The true measure of any society can be found in how it treats its most vulnerable members.” Mahatma Gandhi

The world’s population is aging with the fastest growth in the older population projected to take place in Africa (United Nations, 2019). Likewise, although Ethiopia is known as having a youthful population, the age 60+ population is growing at a faster rate than any other age group. The proportion of older adults in Ethiopia is expected to more than double from 5.1% in 2010 to 10.3% by 2050 (Moges et al., 2014). Rapid population aging in Ethiopia is arriving before the economy, the government, and families are ready to take on the challenges of a growing older population (Moges et al., 2014).

Although large-scale societal trends such as modernization, urbanization, migration, and digitization may contribute to improved life situations for young adults, in Ethiopia, as in other sub-Saharan African nations, these trends are eroding traditional support systems for older adults. As younger family members migrate to cities and towns or out of the country in search of education and better economic opportunities (Wieser et al., 2022), the extended family system that provides care and protection for older adults is breaking down. Kaplan and Inguanzo (2017) explain, “As population aging expands and international birth rates decline, global family structures are going to change significantly, leaving thousands of adults with fewer options for long-term care as they age” (p. 4). Due to changes in family support systems alongside a lack of formal care systems, elder care Africa is viewed by some scholars to be “in crisis” (Freeman, 2023, p. 2).

In an ongoing context of entrenched poverty in Ethiopia, older adults are increasingly viewed as a financial burden. Waning family support, coupled with a lack of social protection (i.e., basic income support) for older adults, is chipping away at the traditional honoring of one’s elders. These cultural shifts are propelling ageism and age discrimination, especially against those older adults who are no longer able to produce an income. Sadly, as a growing number of older adults in Ethiopia lose their livelihood, they are losing their homes and communities.

Although nongovernmental organizations (NGOs) provide some relief for a small proportion of the older population, the Ethiopian government has not prioritized the needs of older adults, clinging instead to a meager residual response. Although the nation has made “considerable progress both in economic growth and social development over the last 10 years … its expenditure on social protection measures has been on the decline” (Teshome, 2013, p. 95). In this paper, we present the *challenges* of the growing older population in Ethiopia, outline some key *changes* that are needed to address those challenges, and consider the *opportunities* that can come about when older adults’ basic needs are met and they are supported in contributing to their communities.

## Challenges of the Growing Older Population in Ethiopia

### Older Adults Not Prioritized in a Context of Economic Instability

Ethiopia is currently facing various challenges including inflation, drought, unemployment, and political instability—all demanding the attention of the government. The overall economic status of the country is impeding the government’s ability to address the needs of all of its citizens. Currently, the national budget and human efforts are being directed to macro-level economic and political issues whereas the majority of older adults lack basic support to meet their daily needs. Many older Ethiopians face both food and housing insecurity. In a study of malnutrition of older adults in eastern Ethiopia, Abdu and colleagues (2020) reported that two thirds suffered from malnutrition or were at risk of malnutrition.

In addition to responding to inflation and unemployment, the government prioritizes the demands of youth and younger adults as their voice of opposition is hostile and even considered dangerous to the governance system. To avoid political turmoil, current government policies and political mottos are tuned to fulfill the interests of the younger population while the basic needs of the older population go unaddressed. The lack of government attention to older adult issues is noted by Woldeyohannis (2018), who reported that factors contributing to food insecurity included a “lack of national guidelines on acute malnutrition management, data on older people not included in regular facility reporting, and older people not routinely included in needs assessments” (p. 3). Sadly, throughout Ethiopia, the needs of the older population are not prioritized.

The rising cost of living in Ethiopia has made it increasingly difficult for many inhabitants, regardless of age, to afford to meet their basic needs for food, clothing, and shelter. For example, in 2010, a kilo of shiro (staple food source) cost 2 Ethiopian birr; nowadays, a kilo of shiro costs 190 birr. The monthly rent for a two-bedroom condo in the Semit area of Addis Ababa increased from 2,500 birr in 2010 to 15,000 birr in 2022. Increases in the cost of living (currently at 39%) have pushed many to leave their rural homesteads looking for ways to fulfill their basic needs, either through work, begging, or other alternatives. The same is true for older adults who were previously able to get their basic needs met within their extended families in their original birthplace (Gebeyaw et al., 2022). Older adults are the primary victims of the increased cost of living as they are left aside by younger relatives who increasingly migrate from rural areas to towns in search of better income (Tegegne & Penker, 2016). Although previously older adults were taken care of by their children or grandchildren, these days older adults can be seen on every street in every town begging or looking for work. According to Teshome (2013), of the more than 100,000 beggars in the capital city of Addis Ababa, the majority are older adults. A growing number of older adults are forced to struggle by themselves to meet their basic needs, especially those with disabilities or whose physical condition does not allow them to do manual labor.

The impact of the increasing cost of living is multifaceted. Not only are fewer people around to help older adults, but there is also a scarcity of basic items needed for survival. Hence, older adults are forced to migrate to the nearest town to escape the growing challenges of life in rural areas. Because the economic struggles are throughout the country, older adults typically cannot get the help they hoped for in their migration destination (Gebeyaw et al., 2022). Even retired civil servants who receive a pension struggle to meet their basic needs because pensions are flat and thus unable to keep up with inflation.

### Declining Respect for Older Adults

Traditionally, older adults in Ethiopia were highly respected, supported by their families, and revered even by people who were not acquainted with them. In times past, older people typically did not leave their own communities because they received full care and support from family members. If older adults left their home areas for various reasons, they could get support and care wherever they went because of their age. These days, however, that sort of courtesy toward older adults is becoming much less common (Chane & Adamek, 2015b). People not only show a lack of support, but sometimes abuse older adults when assertiveness or agility is required to get any service (Zelalem & Kotecho, 2020; Zelalem et al., 2021). Older adults have difficulty securing support services from government and nongovernment institutions. They sometimes need time and calmness to understand and respond to inquiries, and yet service deliverers exhibit little patience in providing information about available services. Hence, older adults in need are discouraged from going to different institutions to request services unless they have someone to accompany them. With no advocate at their side, many older adults experience social isolation, loneliness, and depression, which all may contribute to worsening health issues. If you are not among the few wealthy members of Ethiopian society, in a country with no social protection and no geriatric specialists, your fate is poverty, food insecurity, ill health, age discrimination, social exclusion, and even abuse.

Even though they struggle in their daily lives, many older people in Ethiopia own assets such as houses and plots of land in towns or rural areas. Often, these assets are managed by younger people with the intention of supporting the older asset owners (Chane & Adamek, 2015a). Older adults are considered as dependents, whereas the property and resources they own are used by family members for their own consumption. As older adults in rural areas lose the physical capacity to continue farming, they may reluctantly transfer their agricultural property to younger family members in hopes of receiving ongoing care and support (Mefteh, 2022).

Due to the diminished honoring of older adults over time and the high cost of living, some younger people with connections to older adults find ways to grab their assets. Some adult children with aging parents are not even willing to take them to health facilities when they are sick, longing to inherit the resources when their parents pass away. Sadly, due to the financial constraints across the nation, people are being mistreated as they grow older, especially if they are unable to work. In line with Kwan and Walsh’s (2018) scoping review of global aging and poverty, in Ethiopia as elsewhere, the two primary barriers to older adults being able to contribute to development are poverty and social exclusion.

### Lack of Aging-Specific Services and Policies

In Ethiopia, the lack of proper infrastructure and support services geared toward the older population is a huge obstacle to ensuring older adults’ well-being. By comparison, people with disabilities have recently gained special consideration in accessing various services through government and nongovernment institutions. Due to ageist attitudes of community members, older people are neglected in service delivery institutions. In Ethiopia, as in other sub-Saharan nations, there is a severe shortage of professionals and scholars with geriatric or aging expertise (Naidoo & van Wyk, 2019). Dotchin and colleagues (2013) surveyed sub-Saharan nations and found that most did not have a single geriatrician. A more recent survey of aging scholars in sub-Saharan Africa confirmed that the lack of geriatric professionals and geriatric training was a major challenge to meeting the social and health needs of older adults (Adamek et al., 2021).

Currently, no national policy addresses the older population in Ethiopia. Although a previous administration in Ethiopia endorsed a National Action Plan on Aging (MoLSA, 2006), there has been little action and no financial commitment of the central government to older citizens. Instead of an institutional approach to ensure older adults’ well-being, the Ethiopian government has taken a minimalist residual approach, addressing only the most severe needs of vulnerable older adults. During the coronavirus disease 2019 (COVID-19) pandemic, the government distributed limited food supplies to homeless older adults (Takele et al., 2022) and recently the Addis Ababa city administration initiated a charitable program to feed older adults in the capital city. Likewise in rural areas, the Productive Safety Net Programme reportedly bolstered informal care systems for some older adults, but ultimately this residual approach has had a very limited impact (Alambo & Yimam, 2019). Apart from these charity-based efforts, there is little consideration for older people who are suffering from neglect and hunger across the country.

A few individuals courageously took the initiative and established nongovernmental centers to address the needs of older people, such as “Mekedonia” and “Kibir le Aregawuyan.” However, such facilities are few in number and low on resources and thus are unable to address the full scale of older adults’ needs (Dawud et al., 2021). With a few exceptions such as HelpAge International-Ethiopia (2016, 2018), most NGOs in Ethiopia focus on supportive activities for children, young people, and women. Based on a systematic review of aging research in East Africa, Mussie and colleagues (2022) identified various ethical concerns with the most prominent being the inattention of governments in Africa to older adult issues. Despite the call by the United Nations for universal health care globally (Gebremariam & Sadana, 2019), currently, no sub-Saharan African nations ensure access to geriatric health care.

#### Inaccessibility and digitization of services

The few social services that are available in Ethiopia are not easily accessible to many older adults due to limited mobility and health issues. Services are assumed to serve people of all ages; yet in the absence of advocates for older adults, their needs remain unaddressed. To avoid the public embarrassment and humiliation they are likely to encounter in the process of seeking services, many older adults choose to forego needed health or social services. The overall health situation of many, if not most, older adults deteriorates in the absence of needed care and follow-up. Compounding the barriers to access to social and health services, infrastructures such as roads, transportation, and government buildings are not senior friendly. Roads in many towns do not have pedestrian walkways or are very narrow and hence it is very difficult for many older adults to move from place to place.

In short, the well-being of older people is not given due attention in the Ethiopian context. Despite efforts by the Ethiopian Elders and Pensioners National Association (EEPNA), higher government officials are largely uninformed about the interests and needs of older people (Zerihun et al., 2023). The culture of benefiting from the accumulated experience and wisdom of older people has been declining in Ethiopian culture over time.

With access to many goods and services becoming digital, that is, paying bills, buying and selling, business registration, and application for government services (Senshaw & Twinomurinzi, 2020), a person needs to have a device such as a smartphone, iPad, or laptop that supports digitized systems as well as the knowledge of how to use them. Government-provided services are shifting to online systems and anyone seeking services is expected to go through the required online process. However, no support mechanisms are in place to assist those—including older adults—who are unfamiliar with or do not have digital devices. No training or informative literature is provided to help older adults navigate the current online systems that provide access to services available in the market.

Older adults face difficulty in adapting to this paradigm shift and again are neglected and largely unable to access digital systems. The transformation of many services to digital platforms, along with older adults’ deteriorated health situation, intensifies their poor living conditions. Unless training support is provided to inform older adults how to access available services, older adults will continue to be excluded from services.

## Changes Needed to Address the Challenges

Older adults in Ethiopia are faced with multidimensional challenges that call for collaborative efforts from different stakeholders at local, regional, and national levels. However, some measures should be given the utmost priority: combating negative attitudes toward older people, strengthening geriatric/gerontology and social work education and research, and developing aging-specific policies and services.

### Reframing Aging: Combatting Negative Attitudes Towards Older People

The derogatory attitudes toward older adults in Ethiopia and other sub-Saharan nations, coupled with the decline of intergenerational solidarity, fuel the inequitable access to resources. Unless negative attitudes toward older people are reversed, structural discrimination and supportive policies will continue to exclude older people. The effort to reframe aging in a positive light will take a wide-scale long-term effort involving many stakeholders. Meaningful changes can be achieved through concerted efforts involving most segments and institutions of society. Families, communities, and nongovernmental and faith-based organizations can play a significant role in mobilizing the public to debunk ageist stereotypes and counter negative attitudes toward older adults (Chane & Adamek, 2015a). One misperception is that older people are unproductive, dependent, passive, and a burden to families and the nation’s economy. Older people’s contribution is neither recognized nor valued; thus, getting older is uncelebrated. Negative attitudes toward older adults are based on prejudices, stereotypes, assumptions, and myths about aging and older people. The growing ageism in Ethiopia underscores the need to reframe aging. Valuing older adults and acknowledging their agency is a prerequisite for families, communities, and governing bodies to protect, empower, and care for older adults.

Recognizing older adults’ agency includes involving them in decision-making. To recognize and maintain older adults’ agency and respect their human rights, families and communities must empower older adults to participate in social, economic, civil, and religious life domains, thereby allowing them to contribute to their families, communities, and nation. As active engagement in various life domains helps older adults realize active aging and quality of life (Teshome et al., 2022), opening up leadership positions at different levels to older adults would contribute to the positive reframing of aging. Leadership roles can also provide powerful leverage for older adults to advocate for an equitable share of the nation’s resources.

Involvement is always vital in empowering individuals. Older adults’ active participation in various life domains would not only empower them to contribute to their families and communities but would also demonstrate to younger people that older adults still have much to contribute. By inviting older adults to actively engage in their families and communities, intergenerational solidarity will be strengthened. Furthermore, active engagement will help older adults stave off isolation and loneliness—two common social ills among the older population (Walsh et al., 2020). Due to the ramifications of modernization, there has been an increasing trend to ridicule older people’s capabilities and exclude them from assuming meaningful roles in economic, social, and religious domains, pushing them to the side to lead a sedentary life, leaving their invaluable skills, experience, expertise, and wisdom underutilized.

The media is positioned to play a critical role in a concerted effort to combat ageism in the country. The media can promote the rights of older adults and emphasize the assets they offer (e.g., wisdom, skills, and expertise) and the productive contributions they can make to their families and communities. In doing so, the media should be careful not to unintentionally reinforce the existing ageism. Instead, media outlets can use positive and empowering words in reference to older adults and their needs and capabilities. TV and radio broadcasts can provide education and raise awareness about older people’s rights, needs, and contributions. Different observances can be organized at the national and community levels to honor older adults, recognize their contributions, and raise awareness about their rights, needs, and challenges. For example, the EEPNA sponsors events annually on June 15 in recognition of World Elder Abuse Day (Zerihun et al., 2023). In addition, the recently approved education policy includes moral and ethical education in elementary school which will contribute to shaping the distorted value system of the younger generation in relation to supporting older people.

### Strengthening Geriatrics/Gerontology and Social Work Education and Research

#### Gerontology and geriatrics education and research

Gerontological and geriatric education and research are nearly absent in Ethiopia. Apart from an introduction to gerontology courses focusing on basic knowledge of the discipline being offered in a few Ethiopian universities, neither gerontology nor geriatrics has gained the place and attention they deserve. As is the case throughout sub-Saharan Africa (Buowari, 2023), there is almost no geriatric training and no specialized geriatric care in Ethiopia (Mussie, 2023). The absence of gerontology as the leading discipline in addressing the challenges of aging and the lack of geriatric specialists has created a colossal gap in promoting the social and physical well-being of older adults. With gerontological and geriatric study and education undeveloped in Ethiopia, the deleterious effects of ageism have been able to thrive.

#### Social work education and research

In many nations, the discipline of social work has become increasingly concerned about the well-being of older people and social practice with the older population has continued to grow and change immensely (Berg-Weger & Morley, 2020). In Ethiopia, comparatively speaking, social work education, research, and practice have continued to improve, albeit they are not well established. Social work as a discipline was re-introduced in the country in 2004—just two decades ago—and has yet to be widely accepted as a profession (Gebremariam, 2021). Due to its relatively recent emergence in Ethiopia, social work education has not yet played a significant role in bridging the wide gap in the fight against ageism. However, its contributions to promoting the well-being of older people in the country are not negligible.

Although field education in social work is the cornerstone of academic training, the social work curriculum in Ethiopia reserves a tiny space and resources for field education. Part of the problem is attributed to the distorted image or perceptions that academic committees have of social work education. Many faculty and administrators in Ethiopian universities do not understand how central field education is to successful social work education. As a result, they severely limit the budget for field education. The poor public image of social work at universities is an extension of the general poor public image of social work in Ethiopia.

The intersecting effects of the nascency, distorted image, and low quality of social work education in Ethiopia have resulted in a shortage of professionally competent, dedicated social workers who combat ageism in all its forms, advocate for the rights of older adults, and promote the holistic well-being of older people in every possible avenue, including practice and research. The dearth of aging literature and gerontological studies in Ethiopia conspicuously lays bare the lack of attention to older adult issues (Adamek et al., 2021). Unless universities embark on producing professionally competent, dedicated social workers and other helping professionals with aging expertise, the needs of older adults in Ethiopia will remain unaddressed, and their voices will remain unheard.

##### Enhancing geriatric/gerontology and social work education in Ethiopia

One viable avenue to address the problem is to raise the public image of gerontological, geriatric, and social work professionals. First, the misperception of gerontology at the university level calls for experience sharing between Ethiopian universities and universities in other nations. Providing opportunities for academic staff from Ethiopia to visit schools of social work with established aging programs can help improve attitudes toward the social work profession and especially gerontological social work. However, caution should be taken. We are not suggesting the utter adoption of Western gerontology and social work education curriculum in Ethiopia. There has already been, for instance, a disconnection between social work education curriculum and cultural context in Ethiopia. The curriculum was largely adopted from the social work education in Global North nations without tailoring it to the local context or incorporating indigenous perspectives, knowledge, skills, and values. As scholars such as Crampton (2015) have argued, such a disconnection calls for decolonization of social work in Ethiopia. Thus, experience sharing between Ethiopian universities and Western universities should not result in a wholesale transference of practices, curriculum, and policies that do not reflect local Ethiopian contexts.

Second, establishing a national council on geriatric and gerontology education would significantly strengthen the quality and availability of gerontology/geriatrics education and practice. Third, universities can strengthen their linkage with agencies and organizations that work with and on behalf of older people and other vulnerable populations. Strong connections between universities and these agencies and organizations would give schools an excellent opportunity to raise the public image of both gerontology and social work in partnership with government agencies and organizations advocating for the rights of older adults and promoting their well-being. Collaborating with such agencies and organizations would also create opportunities for students to be employed in these entities, changing the profession’s image, and attracting more people to join the field.

##### Conduct research and comprehensive assessments of the older adult population

The paucity of gerontological studies in Ethiopia is another huge gap that needs due attention to combat the challenges faced by the older population. A comprehensive national survey is needed to document the scope and severity of the older population’s needs and challenges. Most studies on older adults conducted in Ethiopia, despite indicating the nature of the problems older adults are suffering from, do not indicate the scope of the problems and cannot be generalized to the entire older population. The magnitude of older people’s challenges (e.g., poverty, food insecurity, lack of affordable and quality healthcare, and elder abuse) has not been investigated comprehensively through quantitative surveys, and thus, informed policy, programs, and practices cannot be tailored to the overall older population. While conducting comprehensive assessments, the existing disparities between older adults in urban and rural areas of the country should be considered because the two populations have unique needs and challenges. Furthermore, the influences of intersecting factors such as gender, education, social status, and living arrangements on the well-being of older adults need to be explored. In addition to disclosing the magnitude of older adults’ challenging life situations, such comprehensive studies can also significantly sensitize different stakeholders to the needs of older Ethiopians. Regional and interdisciplinary studies of aging in Africa are needed to advance understanding of the impact of societal trends and structural factors influencing the well-being of older adults. Likewise, based on a systematic scoping review of aging-related studies in Africa, Kalu and colleagues (2021) call for collaborative aging research supported by national and international funding agencies.

### Develop Aging-Specific Policies and Services

A leading factor in the deteriorating life situations of older people in Ethiopia is the absence of aging policies tailored to the older population’s needs. The absence of aging policies that address the needs of older people in Ethiopia is chiefly attributed to the widespread negative attitudes toward the population. Unless meaningful policies are established and implemented, older people in Ethiopia, as it stands, will continue to suffer from exclusion and be regarded as a burden to the nation’s economic resources. In the absence of a national aging policy, the social and health ills of older people will not be alleviated. Aging policies/programs would (a) lay the foundation for institutional endeavors to combat the challenges of older adults; (b) institutionalize the responsibility of the government to extend its support to its older population; (c) help lead concerted efforts to address the hardships of older adults; (d) reframe aging, exhibiting that older people deserve care, support, and reverence; and (e) assume a central role in lessening the public’s increasing propensity to regard older people as passive, unproductive, and a burden to society. For aging policies to succeed, they should be comprehensive and universal in scope, requiring no employment eligibility. The vast majority of older people in Ethiopia either work in informal sectors to earn a living or participate in rural labor, including archaic subsistence agriculture. Commitment to a national aging policy is needed to lessen the economic hardships of older people and would be a great stride forward in celebrating aging and honoring Ethiopia’s older citizens.

## Opportunities With an Aging Population

### Assets of Older Adults

So often when population aging is discussed, there is a foreboding tone about the problems and heavy burdens that are expected to arise with a growing proportion of older people. Although an accurate assessment of the needs of a nation’s older adults is a critical first step in policy-making, too often the potential benefits that may come with a greater proportion of older adults in the population are overlooked. How often do nations commission a comprehensive assessment of the assets of their older citizens? In nations where the basic need for social protection of older adults is met and health care is readily available, the majority of older adults are independent, relatively healthy, contributing members of their communities. In nations where older adults have a secure foundation of active aging (health, security, social participation), they are able to thrive and contribute to their families, communities, and nation (%5bAU:WHO, 2002). When a basic foundation of support is in place, later life can be a time of contribution, creativity, and legacy-building. Likewise, if supportive policies were put in place for older Africans, this scenario of active aging could be possible in sub-Saharan Africa (Teshome et al., 2022).

Interestingly, most of the political and community leaders throughout Ethiopia are older men. Yet, the fact that these regional and national leaders are mostly older adults is rarely acknowledged. Unfortunately, this powerful group of older leaders is not viewed as role models of productive aging for other Ethiopians and despite their positions of influence, they rarely advocate for their age peers.

Despite a dearth of governmental advocates for policies to support older adults in Ethiopia, there are nevertheless some examples of older adult leadership of NGOs. At the national level, the Ethiopian Older People and Pensioner’s National Association (OPPNA) advocates for policy changes to support pensioners and other older citizens (Zerihun et al., 2023). Despite its tireless efforts advocating for policies to meet the basic needs of older adults in Ethiopia, OPPNA is meagerly funded and unable to single-handedly turn the tide of policy exclusion.

### Disrupting Ageism

Ageism is defined as “prejudiced attitudes toward aging and older adults that is characterized by myths and stereotypes that depict aging as a process of decay” (Whitsbourne & Sneed, 2002, p. 264). Ageism consists of negative discrimination and prejudice as well as positive stereotypes of older people (Iversen et al., 2009). Excessive care or compassion that stems from perceptions of older people as dependent can also patronize older people, negatively affecting their well-being (Iversen et al., 2009). Ageism can also be self-directed (Levy, 2009) due to internalization of ageist attitudes and stereotypes (Ayalon & Tesch-Römer, 2017). Self-directed ageism may not be considered ageism by older persons. Hence, cultural contexts and older people’s voices should be considered in defining ageism and devising strategies for combating the problem.

The destructiveness of ageism and its tendency to discount older adults and their capacity to contribute to society is evident globally. Ageism is insidious, including in Global North nations that have a well-developed social protection system, a national network of support services for older adults, and strong legal protections against age discrimination. In the U.S. American Association of Retired Persons, a large membership organization that advocates for older adults nationally, has found it necessary to launch an anti-ageism campaign, popularizing the phrase “disrupt ageism” (Jenkins, 2018). Likewise, the National Center to Reframe Aging (NCRA) based in Washington, DC, operates on the assumption that a just society ensures that all citizens, regardless of age, can participate and contribute in meaningful ways. Starting as an initiative in 2012, this organization aims to reframe the nation’s thinking, speaking, and actions related to aging and older adults, recognizing that “ageism harms us all” (www.reframingaging.org). A key message of the NCRA is that limiting opportunities for older adults to contribute is an injustice to people as they age and a loss to communities.

If ageism is alive and well in nations that provide an array of supports for their older citizens, it is not surprising that ageism is a core barrier to policy development around the support of older adults in Global South nations. Despite the Ethiopian government ratifying various international documents relating to human rights, Teshome (2013) points out that in practice, the Ethiopian government tends to operate under the long-standing notion of the community and not the national government as the duty bearer for social protection of citizens. However, there is some evidence of change as the mass media in Ethiopia has helped to raise awareness of social protection as a basic human right (Teshome, 2013).

With a base of income support, geriatric health care, and social support, older adults can contribute to their communities in myriad ways. In nations where ageism is less of an impediment to policy development, there are an array of support programs for older adults including congregate and long-term care housing options, social clubs and peer companion programs and cultural centers, and opportunities for older adults to tutor youth and serve their communities in many capacities.

In Ethiopia, there are some successful small-scale models of elder care and contribution that can be expanded. On the local level, an informal system of insurance known as *iddir* provides protection for financial risks such as crop failure or death (Aredo, 2010; Chane, 2021). Community members contribute to a common savings pool that can be drawn from in times of need. In the absence of formal, public resources, *iddirs* serve as an indigenous arrangement to assist community members. It is common for *iddirs* to be led by older members of the community. Another example of local leadership involving older adults in Ethiopia is the system of restorative justice (Ayalew, 2020). Disputes are settled most often outside of court with input from local leaders who are often older adults. Although older adults have the leadership capacity and wisdom to bring about positive change for older adults in Ethiopia, a critical missing ingredient is the political will to bring forth the necessary resources to enable older adults to contribute to their leadership capacity.

A survey of gerontological scholars in sub-Saharan Africa noted several assets of older adults including bringing wisdom, serving as culture bearers, and overseeing restorative justice in local communities (Adamek et al., 2021). If the assets of older adults were valued in Ethiopian society, a Council of Elders could be formed at local, regional, and even national levels to contribute to the resolution of the nation’s challenges—whether those challenges involve the growing older population or other sectors of the population. With input and support from older volunteers, effective indigenous social welfare models could be replicated across the country. As an example, one NGO operating in the capital city of Addis Ababa, known as “Elevating Orphans/Bring Love In,” creates families by pairing widows with orphans and providing housing, education, and basic sustenance (Morris, 2012). In this model, the needs of two vulnerable populations—both widows and orphans—are supported.

In the absence of social programs catering to older people and reliable long-term care, community-based care plays a sizable role in protecting, safeguarding, and caring for older adults. At the local level, families, community, and faith-based organizations, in partnership with various government agencies, NGOs, and older people’s associations, can establish community-based care for older people helping them to age in place. For older adults lacking family support, the Awramba model of community-based care provides an opportunity for rural older adults to be embraced and cared for in a community setting despite any physical or mental limitations (Adamek & Setegn, 2019; Mengesha et al., 2015). When long-term care is needed, there are a few existing models in Ethiopia (Dawud et al., 2021), though hardly enough to meet the growing demand for long-term care. Freeman (2023) cautions against adopting Western designs of long-term care systems, and instead recommends developing systems that fit the African context.

## Conclusion

As throughout much of sub-Saharan Africa, older adults in Ethiopia are increasingly facing lives of poverty and social exclusion. The ageist view of older adults as a noncontributing sector of the population is the real burden in Ethiopia, resulting in a “poverty of policy” regarding late-life issues. In the absence of a basic social protection plan, older adults will increasingly wind up living on the streets and thus the aging population will indeed seem a burden. It is not that older adults in Ethiopia have nothing to contribute and are doomed to decline. Instead, over time they are steadily losing their stature as valuable members of Ethiopian society. When younger family members migrate from rural areas or take over older adults’ assets, older adults are left to fend for themselves (Gebeyaw et al., 2022).

A revival of respect for older citizens is needed to change the tide of public opinion about older adults and to promote supportive policies. As Teshome and colleagues (2022) argue, “Instead of portraying older adults as passive, needy, and dependent, narratives about aging in Global South nations must be transformed so that older adults are viewed as assets and contributors to their families and communities” (p. 18). Likewise, Gebremariam and Sadana (2019) argue that the moral imperative for universal health care for older adults stems from “an understanding of older persons as active agents in the social structure of (their) well-being” (p. 1). Without a transformation in the public perspective of older adults, older Ethiopians—except perhaps the most wealthy—will be doomed to an undignified late life.

Although the challenges faced by older adults in Ethiopia seem like a problem of aging compounded by poverty, the root source of the problem is a *poverty of policy* fueled by ageism. If income support and appropriate health care were provided to older adults, health and well-being in late life would improve, food insecurity and housing insecurity among older adults would lessen, and all Ethiopians could look forward to a dignified late life. The current trajectory of older adults’ lives in Ethiopia is unacceptable. The government of Ethiopia and the nation as a whole must take meaningful action toward honoring older adults by providing social protection and welcoming their contributions, thus bolstering the stability, humanity, and compassion of Ethiopian society, benefiting citizens of all ages.
